# Photo-Oxidative Stress-Driven Mutagenesis and Adaptive Evolution on the Marine Diatom *Phaeodactylum tricornutum* for Enhanced Carotenoid Accumulation

**DOI:** 10.3390/md13106138

**Published:** 2015-09-29

**Authors:** Zhiqian Yi, Maonian Xu, Manuela Magnusdottir, Yuetuan Zhang, Sigurdur Brynjolfsson, Weiqi Fu

**Affiliations:** 1 Center for Systems Biology and Faculty of Industrial Engineering, Mechanical Engineering and Computer Science, School of Engineering and Natural Sciences, University of Iceland, Reykjavík 101, Iceland; E-Mails: zhy1@hi.is (Z.Y.); mam5@hi.is (M.M.); yuetuan.zhang@helsinki.fi (Y.Z.); sb@hi.is (S.B.); 2 Division of Science and Math, New York University Abu Dhabi, and Center for Genomics and Systems Biology (CGSB), New York University Abu Dhabi Institute, Abu Dhabi 129188, UAE; 3 Biomedical Center and Department of Life and Environmental Sciences, University of Iceland, Reykjavík 101, Iceland; 4 Faculty of Pharmaceutical Sciences, University of Iceland, Hagi, Hofsvallagata 53, Reykjavik IS-107, Iceland; E-Mail: xum1@hi.is; 5 Department of Food and Environmental Sciences, Division of Food Chemistry, University of Helsinki, Helsinki FI-00014, Finland

**Keywords:** *Phaeodactylum tricornutum*, UV mutagenesis, adaptive laboratory evolution (ALE), fucoxanthin, neutral lipids

## Abstract

Marine diatoms have recently gained much attention as they are expected to be a promising resource for sustainable production of bioactive compounds such as carotenoids and biofuels as a future clean energy solution. To develop photosynthetic cell factories, it is important to improve diatoms for value-added products. In this study, we utilized UVC radiation to induce mutations in the marine diatom *Phaeodactylum tricornutum* and screened strains with enhanced accumulation of neutral lipids and carotenoids. Adaptive laboratory evolution (ALE) was also used in parallel to develop altered phenotypic and biological functions in *P. tricornutum* and it was reported for the first time that ALE was successfully applied on diatoms for the enhancement of growth performance and productivity of value-added carotenoids to date. Liquid chromatography-mass spectrometry (LC-MS) was utilized to study the composition of major pigments in the wild type *P. tricornutum*, UV mutants and ALE strains. UVC radiated strains exhibited higher accumulation of fucoxanthin as well as neutral lipids compared to their wild type counterpart. In addition to UV mutagenesis, *P. tricornutum* strains developed by ALE also yielded enhanced biomass production and fucoxanthin accumulation under combined red and blue light. In short, both UV mutagenesis and ALE appeared as an effective approach to developing desired phenotypes in the marine diatoms via electromagnetic radiation-induced oxidative stress.

## 1. Introduction

Diatoms form a major lineage of unicellular algae and play an essential role in marine ecosystems [1]. Diatoms account for the production of a large portion of the total energy of the oceans and are also responsible for the global silicon cycle [2]. They have been receiving increasing levels of attention as promising feedstocks for sustainable production of biofuels, pharmaceutical ingredients, cosmetics, and food. The marine pennate diatom *Phaeodactylum tricornutum* is a model species to study diatom physiology and diatom-based biotechnological applications as its genome has been sequenced, annotated and published [1]. In addition, this particular diatom, *i.e.*, *P. tricornutum*, usually exists in three different morphotypes in liquid cultures: fusiform, oval, and triradiate, making it an ideal model to study cellular mechanisms involved in morphological transformation [3]. It grows rapidly and accumulates a large amount of neutral lipids, which can be converted to biodiesel, accounting for as much as 20% of its total dry weight in normal non-stress growth conditions [4].

Fucoxanthin, one type of xanthophyll pigment, is the major carotenoid in *P. tricornutum*, and works as an accessory pigment in chloroplasts together with chlorophyll *a* and *c* forming a complex with the chlorophyll *a*/*c* binding proteins (FCPs) [5,6]. Fucoxanthin absorbs blue-green to yellow-green parts of the light spectrum; the golden brown or olive-green color of diatoms is due to a high amount of fucoxanthin [7]. Fucoxanthin exhibits varied bioactive properties acting as an antioxidant and chemo preventive agent against obesity, cancer, inflammation, angiogenesis, and diabetes; it exerts protective effects on numerous organs such as skin, liver, brain blood vessel, bones, and eyes [7].

To develop diatom-based “cell factories”, and to produce bio-based commodities including biofuels and value-added products such as carotenoids, multiple approaches and strategies have been established to harness diatom’s potential as a feedstock [8]. Nutrient depletions such as nitrogen and phosphate deficiency lead to the accumulation of neutral lipids in diatom *P. tricornutum* [4,9]. Using both meganucleases and TALE (transcription activator-like effector) nucleases to precisely and stably modify the genome of *P. tricornutum*, investigators have been able to enhance triacylglycerol accumulation by 45-fold through disrupting the UDP-glucose pyrophosphorylase [10]. In green microalgae and diatoms, accumulation of carotenoids has been closely linked to the growth conditions such as light quality, salinity, pH, and nitrate concentration. Optimization of these growth parameters can promote high-levels of carotenoid production [11,12,13].

Mutagenesis and selection of mutants has been utilized as an effective approach to increase strain performance of microbes for decades [8]. Algal mutants could be obtained by physical mutagens such as UV and gamma radiations and by chemical mutagens such as EMS (ethyl methanesulfonate) and NTG (*N*-methyl-*N*′-nitro-*N*-nitrosoguanidine) for enhancing the production of carotenoids or biomass [8,14]. Ultraviolet (UV) light has a potent genotoxic effect that induces DNA damage, promotes mutations, and can even cause cancer in animals [15]. UV induced random mutagenesis has an advantage of not being classified as a genetically modified method since in many countries as the ones in European Union (EU) genetically modified organisms (GMOs) may encounter regulatory hurdles [16]. UV light can be generally divided into three categories based on its spectrum: UVA (from 320 to 400 nm), UVB (from 290 to 320 nm), and UVC (<290 nm). UV light causes specific DNA damage such as pyrimidine pyrimidone photoproducts (64PPs) and cyclobutane pyrimidine dimers (CPDs) [15]. The wavelength resulting in the highest formation of 64PP and CPD is around 260 nm, which is the same as the peak absorption wavelength of DNA. Consequently, UVC promotes more DNA damage and more random mutations than UVA and UVB. Therefore, UVC would be a suitable radiation source to mutate *P. tricornutum* cells. In this study, *P. tricornutum* cells were exposed to UVC light, subsequently followed with 96-well microplate selection. Strains selected from UV radiation were studied and the major pigments and neutral lipids in cells were analyzed.

**Figure 1:**
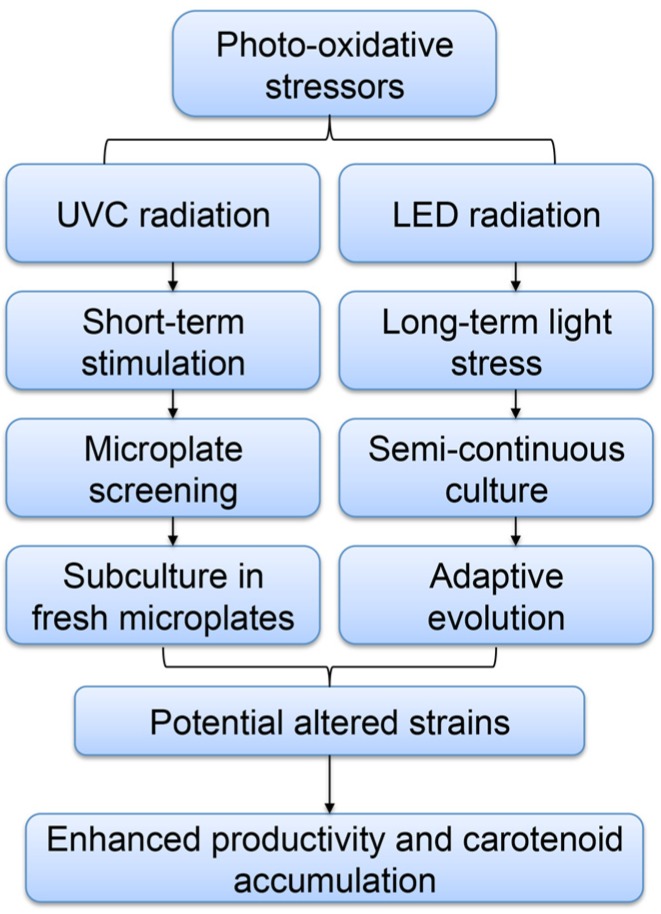
Schematic process for improving diatoms for value-added products.

Adaptive laboratory evolution (ALE) has been established to develop new phenotypic and biological functions and improve strain performance [17]. It has been proven successful in improving strain function in adaptation to specific conditions and enhancing their tolerance to abiotic stresses in different green algal species [18,19]. However, no study has been reported on applying ALE in diatoms. We have set out for the first time to utilize ALE to improve both growth performance and carotenoid accumulation without compromising growth for high-level carotenoid productivity in *P. tricornutum* culture. Schematic process was shown in Figure 1.

## 2. Results and Discussion

### 2.1. Screening of P. tricornutum Mutants

**Figure 2:**
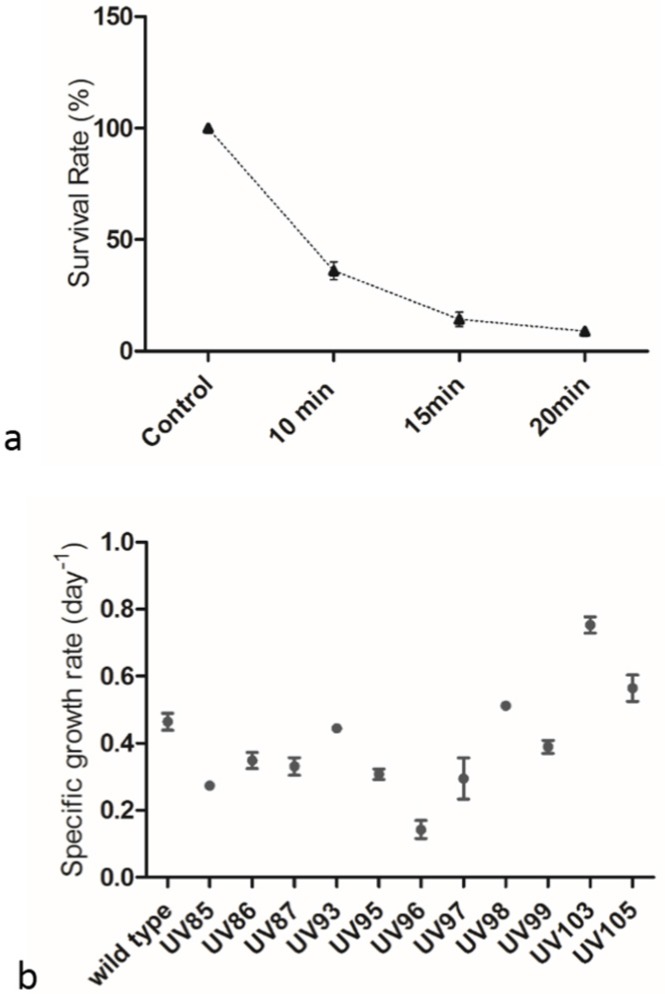
(**a**) The lethality curve of *P. tricornutum* under different UV exposure times; (**b**) The growth of screened UVC treated strains. Each well of strain had been sub-cultured in biological triplicates and each triplicate had been technically measured twice. The seed culture of wild type for comparison was taken from an Erlenmeyer flask culture under logarithmic growth.

The lethality effect of UV exposure time was studied by subjecting *P. tricornutum* to UVC radiation for different time periods. The survival rate was dependent on the UV exposure time, and the prolonged exposure time led to lower survival rate (Figure 2a), which was consistent with previous publications [14,16,20]. It was found that 15.0 min of UV exposure time resulted in an approximately 14.4% survival rate while 36% and 8.9% survival rates were achieved upon 10.0 min and 20.0 min UV treatments, respectively (Figure 2a). To increase the chance of obtaining mutants, exposure times of 10.0 min and 15.0 min were chosen for further UV mutagenesis experiments. A total of 11 mini-pools of strains that showed a higher growth rate which were seeded in twenty 96-well microplates were selected and re-cultivated in 48-well microplates. Mutants designated as UV85 to UV99 were exposed to UVC for 15.0 min while UV103 and UV105 were treated for 10.0 min (Figure 2b). UVC irradiation is known to induce random mutagenesis in microbes [15,16], and mutants clearly displayed varied growth rates (Figure 2b). In comparison with wild type, UV103 and UV105 had higher specific growth rates, UV93, UV98, and UV99 had approximately identical growth rates, and the rest of the strains exhibited lower growth rates. On average, the specific growth rate for 15.0 min UVC treated strains was 0.34 day^−1^ while the growth rate for 10.0 min UVC treated strains was 0.66 day^−1^. The specific growth rate of wild type was 0.46 day^−1^, implying that long UVC exposure may induce serious growth suppression on diatoms and the cells may exhibit reduced growth performance after recovering from UVC damage.

### 2.2. UVC Treatment Induced Accumulation of Neutral Lipids

**Figure 3:**
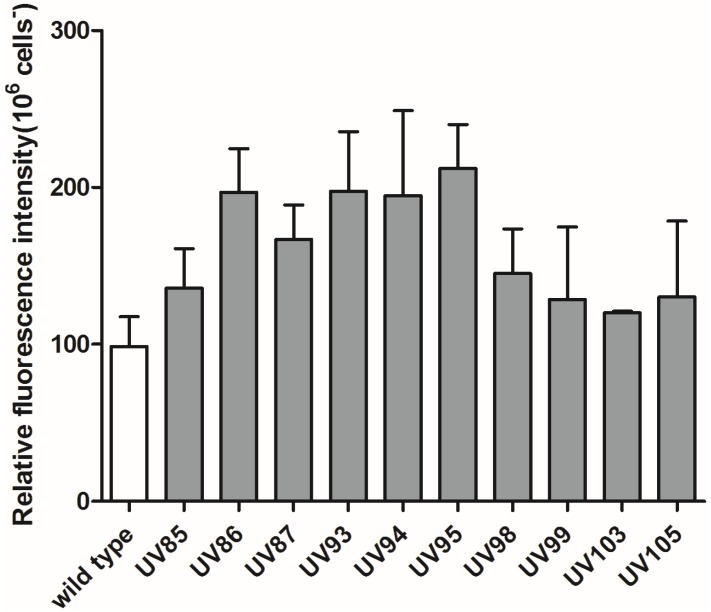
Analysis of neutral lipids in UVC treated strains using Nile red based fluorescence. Strains were incubated with Nile Red (Details in Experimental Design and Methods) in a dark room for 20 min and fluorescence intensities were measured. The intensity differences between stained samples and unstained samples correlate with the content of neutral lipid.

Nine of the eleven selected mini-pool strains were studied further and most UVC treated strains gained enhanced neutral lipid contents than the wild type (Figure 3). UV85 to UV99 were exposed to UVC radiation for 15.0 min, and UV103 and UV105 were exposed for 10.0 min. Strains UV86, UV93, UV94, and UV95 reached approximately two folds of the amount of the neutral lipid content in wild type. According to the gravimetric method (See details in Experimental Section), the neutral lipid content in the wild type was approximately 23.3% of the total dry cell weight (DCW). Therefore, total neutral lipids content in mutants may reach up to 50% of dry weight. On average, 15.0 min UVC irradiated strains accumulated 42.6% of the dry weight while 10.0 min UVC treated strains achieved 34.8% of dry weight as neutral lipids.

Neutral lipids in microalgae that can be easily converted to biodiesel have gained much attention as an alternative to fossil diesel [4]. Previous research has indicated that neutral lipid content increases under several abiotic stresses such as nitrogen deprivation, phosphorus limitation, salinity stress and light stress [4,21,22]. High level accumulation of fatty acids was induced in microalga *Tetraselmis* sp. under stress by UVC radiation [23], so the accumulation of neutral lipids may be recognized as an indicator of cells under stress conditions. In this study, the increased lipid accumulation in *P. tricornutum* cells as demonstrated by enhanced Nile Red fluorescence intensity may be attributed to the mutations generated by the UVC radiation. Strains in 15.0 min UV exposure group had accumulated more neutral lipids, *i.e.*, 42.6% in average than strains in 10.0 min UV exposure group with an average content of neutral lipids of 34.8% while the average specific growth rates were reversed, *i.e.*, higher in the 10.0 min UV treated group (0.66 day^−1^) than in the 15.0 min UV treated group (0.34 day^−1^). Combining these data with the fact that neutral lipids usually accumulate under stress conditions in algae [22], it appears that oxidative stress caused by UVC may also contribute to the increased accumulation of neutral lipids. It has been reported that applying a low dosage of UVC radiation over 24 h increased lipid droplets amount in both *D. salina* and *H. pluvialis* [23], and this study shows that short time and high dose UVC exposure can also accumulate neutral lipids.

### 2.3. Carotenoids and Chlorophyll a Contents in Wild Type P. tricornutum and its UV-Mutants

One of the major species of carotenoids in diatoms is fucoxanthin [7]. It was tentatively identified by both chromatographic and mass spectroscopy information in comparison with reference values from a previous study [24]. Figure S1 indicated that retention times of major fucoxanthin ions were 6.11 min and 6.29 min, comparable to the reference value at 6.36 min as suggested previously [24]. The base peak in the mass spectra of fucoxanthin presented a mass to charge ratio (*m*/*z*) of 641.4192 identified as [M + H − H_2_O]^+^, which suggested the depletion of a H_2_O molecule from the protonated mother ion with a *m*/*z* of 659.4135. Further fragmentation eliminating a molecule of CH_3_COOH gave rise to a product ion at *m*/*z* = 581.3992.

**Figure 4:**
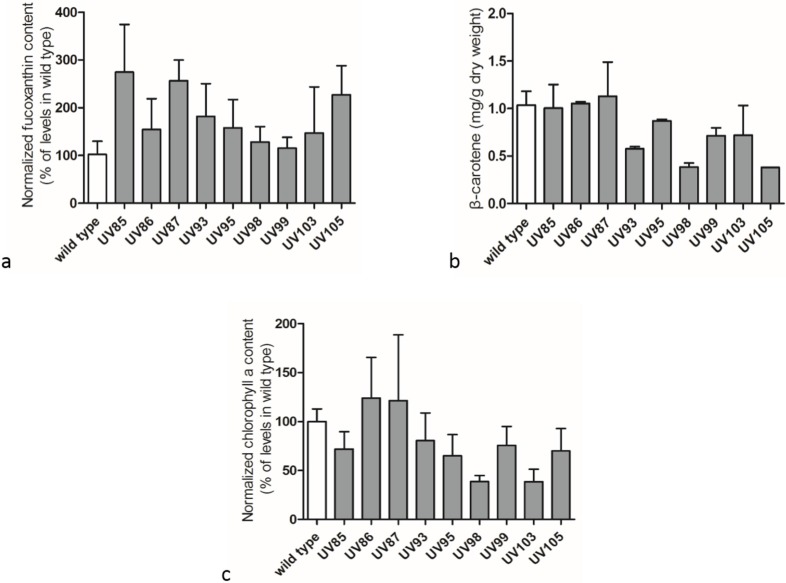
Comparison of fucoxanthin, β-carotene and chlorophyll *a* contents between wild type cells and UV-mutants. (**a**) Fucoxanthin; (**b**) β-carotene; (**c**) chlorophyll *a*. Data were averaged from biological triplicates; error bars represent standard deviation.

Production of carotenoids and chlorophyll *a* in wild type cells and mutants are shown in Figure 4. Beta-carotene was quantified with standards with known concentrations to ensure the quality of extraction and LC-MS analysis processes. The relative fucoxanthin and chlorophyll *a* contents in UV-treated mutants were normalized to the original levels in wild type cells. UV radiation stimulated the accumulation of fucoxanthin in most selected mutants (Figure 4a), and the top strain UV85 yielded an increase of 1.7 times of fucoxanthin content in comparison with the wild type cells. However, β-carotene and chlorophyll *a* contents (Figure 4b,c) decreased in some UV-mutants. In these mutants, it appeared that the increase in accumulation of neutral lipids was positively correlated with the enhancement of fucoxanthin production in cells though the correlation was not proportional. It has been found that every selected mutant had higher fucoxanthin content than the wild type. This phenomenon may be explained as that fucoxanthin plays an essential role in coping with photo-oxidative stress [7] and could be directly induced with light stress treatment. The autogenous up-regulation of fucoxanthin might offer a better defense barrier against photo-damaging effects. It is likely that the majority of the mutants exhibiting elevated fucoxanthin level were induced by UVC oxidative stress and only a small fraction of mutants were mediated through causative mutations. The fucoxanthin enhancement could be protective to *P. tricornutum* cells against UVC exposure. Other antioxidant molecules such as astaxanthin and docosahexaenoic acid (DHA) might also be actively induced to protect cells against oxidative stress. In addition, antioxidant enzymes such as superoxide dismutase (SOD), glutathione reductase (GR), and ascorbate peroxidase (APX) that play important roles in defending the oxidative stresses might be induced under UVC radiation in *P. tricornutum* as well [25]. Therefore the UVC radiation approach in this study may be useful in developing a series of strains that accumulate particular antioxidants in defending againt oxidative stress for value-added products.

### 2.4. Adaptive Laboratory Evolution (ALE) Increased Growth Rate and Fucoxanthin Content

**Figure 5:**
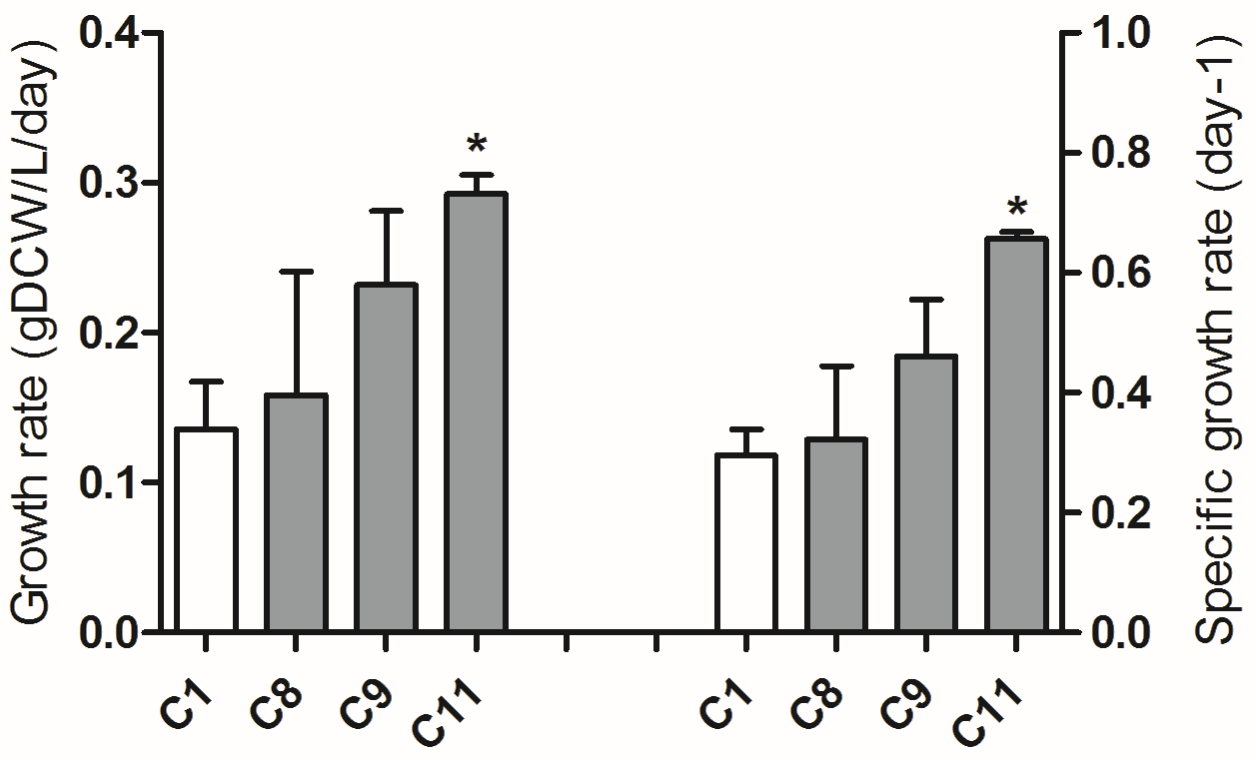
*P. tricornutum* growth rate and specific growth rate over cycles during adaptive laboratory evolution (ALE). The average growth rates correspond to biomass produced per day in one cycle. The results were averaged from three biological replicates and error bars represent standard deviation. Asterisk represents statistically significant difference between C11 and C1 (*p* < 0.05).

**Figure 6:**
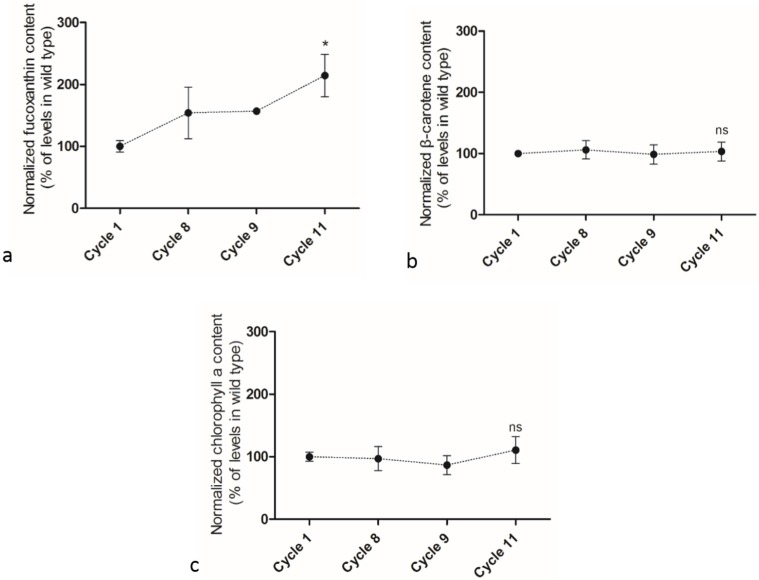
Changes of fucoxanthin, β-carotene and chlorophyll *a* contents during adaptive laboratory evolution. (**a**) fucoxanthin; (**b**) β-carotene; (**c**) chlorophyll *a*. Data were averaged from three biological replicate and error bars represented standard deviation. Asterisk means significant difference (*p* < 0.05) and “ns” indicates no significant difference between C11 and C1.

Adaptive laboratory evolution (ALE) was performed in a semi-continuous culture mode and cell densities were kept the same at the beginning of each ALE cycle by removing part of diatom culture and adding fresh medium [17]. After completing 12 cycles of ALE, it was found that the growth rates at Cycle 12 showed no significant difference in comparison with that at Cycle 11 (Supplementary Figure S2). Then only samples from the 11 cycles of ALE were studied in detail. The growth rate of *P. tricornutum* cells increased gradually over ALE cycles, from a rate of 0.14 gDCW/L/day at Cycle 1 to 0.29 gDCW/L/day at Cycle 11 (Figure 5). The neutral lipid content in cells slightly decreased from 24.6% at Cycle 1 to 19.3% at Cycle 11 (Supplementary Figure S3). After 11 cycles of ALE, β-carotene, fucoxanthin, and chlorophyll *a* contents were measured by UPLC-UV-MS (See details in Experimental Section). Both chlorophyll *a* and β-carotene did not show any significant changes during the whole ALE process while the fucoxanthin content in cells increased progressively (Figure 6). The fucoxanthin content at the end of the 11th ALE cycle was 2.1 times of initial level at the first cycle.

Adaptive laboratory evolution has been developed and used during the last few decades to adapt microorganisms to defined conditions [17]. ALE promotes phenotype changes and the strains’ adaptation to environment therefore improves the strains’ performance via particular metabolic pathways and concentrates energy assimilated on growth through down-regulation of stress-response pathway or defense pathway [17,26]. Light emitting diodes (LEDs) that offers longer lifetimes and higher energy efficiency are becoming one of the most prevalent light sources in the world [27] and the quality LEDs that emit narrow spectra may be an effective tool to study light effects on green algae as well as diatoms [28]. It has been reported that ALE combined with LED has successfully enhanced the accumulation of carotenoids and the growth rate in green microalgae *C. vulgaris* and *Dunaliella salina* [13,18,19]*.* The average LEDs intensity in present study was commanded through the duration of the on/off cycle (0.1 ms) [18]. Since the peak light intensity of 250 μmol m^−2^s^−1^ was much higher than the average intensity [12,18,19], this flash light device is likely to have produced additional light-induced oxidative stress to the ALE samples.

It is the first time that ALE has been manipulated on diatoms in this study. As a fundamental structural pigment, chlorophyll *a* was maintained stable during the whole ALE process, consistent with the ALE results reported for *D. salina* [18]*.* The strains had already been cultivated in the PBRs for one week before ALE started. The final fucoxanthin content was approximately two-fold higher than the initial level and the final growth rate was also approximately two-fold higher. The mechanisms to synthesize and accumulate large amounts of fucoxanthin are still not clear. Elevated light stress accumulated carotenoid content in *D. salina* and *C. vulgaris* [18,19], and it may be speculated that light-induced oxidative stress also plays an important role in the fucoxanthin biosynthesis in *P. tricornutum* in this ALE process. It is possible that adapted strains with increased fucoxanthin content might have higher light utilization efficiency, consequently improving the growth rate. On the contrary, the neutral lipid content decreased slightly, from 24.6% at Cycle 1 to 19.3% at Cycle 11. Linking the UV growth rate and neutral lipid results, it implies that neutral lipid content is not positively correlated with growth rate as well as fucoxanthin content. The strains with improved growth performance and fucoxanthin accumulation may come from the metabolic up-regulation of accessory pigments as well as the aggregative mutants created during the ALE process that may be fixed over time [17,18], though the latter is likely to be the minor contributor as no more than 20 generations were achieved in diatoms over 11 cycles. The results showed that adaptive laboratory evolution is an efficient method to promote both carotenoids accumulation and growth rate in the marine diatom *P. tricornutum*. A combination of UV mutagenesis and ALE could also be a promising approach in improving strain properties.

## 3. Experimental Design and Methods

### 3.1. Diatom Culture and Growth Conditions

*Phaeodactylum tricornutum* strain (CCAP 1055/1) was purchased from the Culture Collection of Algae and Protozoa (CCAP), Scotland, U.K. The cultures were grown at 22 ± 2 °C in the modified f/2 medium without silicate in which additional 10 mM nitrate and 3 mM magnesium sulfate were added. Culture pH was maintained at 8.0 ± 0.5, unless otherwise indicated. For Erlenmeyer flask and microplate cultivation, continuous illumination was provided by the fluorescent lamps with a light intensity of 25 μE/m^2^/s as measured with a quantum sensor (SR. NO. Q40526 of QUANTUM, Model LI-1400, LI-COR biosciences, Lincoln, NE, USA) on the surface of an empty Erlenmeyer flask and microplate. To ensure the diatom cultures were maintained under exponential growth phase, the initial concentration was at an optical density of OD_600_ = 0.1 ± 0.05 and the final density was controlled under an OD_600_ of 0.9.

### 3.2. UV Mutagenesis

The ultraviolet C (UVC) exposure was selected as the mutagenic agent (Figure 1). The germicidal tubular UV lamp was purchased from Light Tech (Stock Code: LTC30T8, USA) and the UV Output power was 13.4 Watts with a peak wavelength of 254 nm. Ten milliliters of *P. tricornutum* culture in exponential growth phase with a cell concentration of 5 × 10^6^ cells/mL were distributed evenly on a round petri dish. The cultures were then directly exposed to UV lamp at a distance of 20.0 cm for either 10 or 15 min. Cultures after UV radiation were immediately kept at dark conditions over night to prevent photo reactivation and then transferred to 96-well microplates for growth experiments. The OD_600_ for each well was measured every 24 h by SpectraMax M3 Multi-mode Microplate Reader (Molecular devices, Sunnyvale, CA, USA) to evaluate the growth rate. For all wells in a 96-well microplate, OD_600_ decreased at the beginning and most wells did not show any significant growth after one week cultivation. Eleven mini-pools of strains that had relatively higher growth rates were selected from twenty 96-well microplates and successfully transferred and re-cultured in 48-well plates in triplicate for 4.0 days prior to growth measurements.

### 3.3. Growth Measurements and Calculations

The cell number was counted using Leica DMIRB microscopy and bright-line hemacytometer (Hausser Scientific, Horsham, UK) every 48 h in triplicates and cell concentration (cells/mL) was used to calculate growth rate. The biomass dry weights were measured by collecting cell sample suspension (usually 10.0 mL) on a cellulose membrane (pore size, 0.45 μm), washing samples with deionized water twice, and drying the collected samples at 60 °C overnight before weighing. The specific growth rate is the growth rate related to the population size, and specific growth rate μ = ln (N2/N1)/Δt, where N2 and N1 are the final cell number and the initial cell number, respectively and Δt represents the interval time.

### 3.4. Gravimetric Method for Determining Total Neutral Lipid Content

Fifty milliliters diatom cultures were centrifuged at 4000× *g* for 10.0 min and cell pellets were collected by removing the supernatant. Cell pellets were re-suspended with 4 mL methanol and chloroform (1:1 v:v) thoroughly and sonicated 20 min in an ice bathing. After sonication, 1.0 mL of 5.0% NaCl saline was added and mixed by vortex for one minute. The mixture was then centrifuged at 2000× *g* for 10 min and the lower layer was collected. The extraction process was repeated twice with additional 2 mL chloroform added each time. Total chloroform layers were rotary evaporated in a pre-weighted glass tube by miVac Quattro (Genevac, Ipswich, England). Differences between the after-weighted and pre-weighted were counted as the neutral lipid mass in these cell cultures.

### 3.5. Nile Red Staining for Neutral Lipid Detection

Two microliters Nile Red (0.1 mg/mL in acetone) was added into 200 μL diatom samples in 96-well microplates, mixed properly and Samples were incubated for 20 min at room temperature in dark room. A SpectraMax M3 Multi-mode Microplate Reader (Molecular Devices, Sunnyvale, CA, USA) was used to measure fluorescence intensity of each well. The excitation wavelength and emission wavelength were set as 530 nm and 580 nm, respectively. Fluorescence intensity was detected immediately after 30 s of vigorous shaking. The relative fluorescence difference was utilized to screen different strains according to the neutral lipid content in each stain. All samples were prepared in triplicates.

### 3.6. Adaptive Laboratory Evolution (ALE)

Seed cultures for ALE were original wild type and taken from Erlenmeyer flasks under exponential growth phase. Before the initiation of ALE cycle, strains were cultivated in PBRs for one week in order to get preliminary adaptation to the new growth conditions and achieve desired cell density. ALE was implemented with a five-day cycle by a semi-continuous culture system [18,19]. The initial biomass density at beginning of each cycle was set at a fixed density (approximately 0.7 gDCW/L) by removing part of the culture and refilling with fresh medium. The initial and final optical density at 600 nm was measured for each cycle. Bubble cylindrical column photobioreactors (PBRs) were with 30 cm height, 4 cm diameter, 300 ± 5 mL working volume and input gas was 135 mL/min air with 1% CO_2_ [18,19].

### 3.7. Artificial Light Setup

The artificial light supply was setup with 75% red LED light (Part number: SSL-LX5093SRC, LUMEX, Taiwan) and 25% blue LED light (Part number: VAOL-5LSBY2, LUMEX, Taiwan) with a photon flux of 30.0 μE/m^2^/s based on (Al, Ga) InPsystem. The average photon flux was provided with flashing light at a duty cycle of 12% and at a frequency of 10 kHz [18,19]. The red and blue lights were centered at 660 nm and 470 nm respectively with 20 nm bandwidth at half peak height for both output spectra.

### 3.8. Chlorophyll and Carotenoid Analysis

The procedures used here were described previously [24,29]. An aliquot of 0.50 mL cell culture was centrifuged at 2000× *g* for 10.0 min. The cell pellet was collected and re-suspended with 3.0 mL of ethanol and hexane mixture (2:1 v/v) containing 0.1% butylated hydroxytoluene by vortex mixing until the solution was colorless followed by the addition of 2.0 mL of de-ionized water and 4.0 mL of hexane. The samples were vigorously mixed and the mixture was centrifuged again at 2000× *g* for 5.0 min. An aliquot of upper hexane layer was transferred to another glass tube, evaporated with miVac Quattro (Genevac, England) at room temperature and re-dissolved with methyl tertiary butyl ether (MTBE): acetonitrile (ACN) (1:1 v/v). An aliquot of 5.0 μL sample was taken and analyzed by ultra-high performance liquid chromatography, coupled with UV and mass spectrometer (UPLC-UV-MS) [24,29].

UPLC separation was performed on ACQUITY UPLC (Waters, Milford, USA) using an HSS T3 1.8 μm column (2.1 × 150 mm; Waters, UK) by reversed phase chromatography. The mobile phases constituted Phase A: ACN+methanol+MTBE (7:2:1, v/v/v) and Phase B: 10 mM ammonium acetate. Elution flow rate was kept consistent at 0.45 mL/min and with a gradient of 60% Phase A at 0 min, 75% Phase A at 4.0 min, 100% Phase A at 12.0 min, 98% Phase A at 22.0 min, 60% Phase A from 23.0 and 27.0 min. UV detection was carried out by a TUV detector (Waters, Milford, USA) at 450 nm.

## 4. Conclusions

In this study we explored photochemically induced mutagenesis and adaptive laboratory evolution on the growth and pigment accumulation in the marine diatom *P. tricornutum*. UVC induced stress-driven mutagenesis enhanced neutral lipids and fucoxanthin accumulation and created mutants with different growth rates. Adaptive laboratory evolution (ALE) promoted both growth performance and fucoxanthin levels in *P. tricornutum*. These results indicated that both methods may be promising tools in modifying diatoms for the accumulation of value-added carotenoids. UVC mutagenesis and ALE may be combined as an effective strategy in further developing *P. tricornutum* strains for industrial applications.

## Supplementary Files

Supplementary File 1
