# Supplementary material for: Photo-Oxidative Stress-Driven Mutagenesis and Adaptive Evolution on the Marine Diatom Phaeodactylum tricornutum for Enhanced Carotenoid Accumulation
Source: Mar Drugs. 2015 Sep 29;13(10):6138–51. doi: 10.3390/md13106138 (PMC4626683; doi:10.3390/md13106138)
Supplement: Supplementary File 1 [file marinedrugs-13-06138-s001.docx]

Supplementary Information


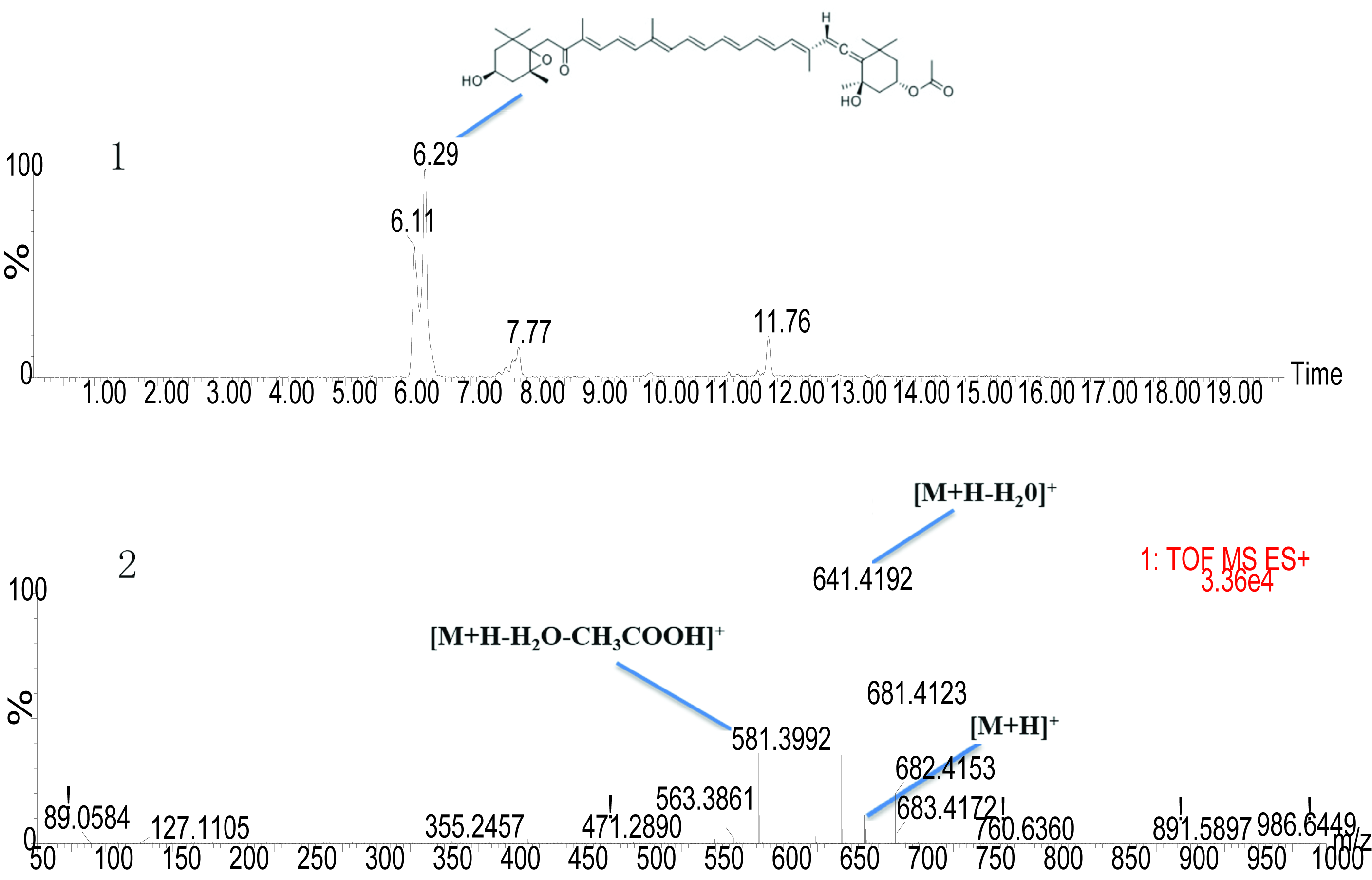


**Figure S1.** Identification of fucoxanthin by LC-MS [1]: (1) mass spectra of fucoxanthin eluted out at 6.29 min; (2) extracted ion chromatogram at *m*/*z* 641.4.

**Figure S2.** Comparison of growth rates in *P. tricornutum* cells between Cycle 11 and
Cycle 12 during adaptive laboratory evolution (ALE). The growth rates correspond to biomass produced per day in average in one cycle. The results were averaged from three biological replicates and error bars represent standard deviation. ns represents no statistically difference (*p* > 0.05).

**Figure S3.** Effects of ALE on the accumulation of neutral lipids in cells. The neutral lipid content was measured by gravimetric method discussed in the Experimental Section. Each value was averaged from three triplicate experiments. The error bar represents the standard deviation.

Reference

1. Pacini, T.; Fu, W.; Gudmundsson, S.; Chiaravalle, A.E.; Brynjolfson, S.; Palsson, B.O.; Astarita, G.; Paglia, G. Multidimensional analytical approach based on UHPLC-UV-ion mobility-MS for the screening of natural pigments. *Anal. Chem.* **2015**, *87*, 2593–2599.

© 2015 by the authors; licensee MDPI, Basel, Switzerland. This article is an open access article distributed under the terms and conditions of the Creative Commons Attribution license (http://creativecommons.org/licenses/by/4.0/).
